# Expression of Dual-Specificity Phosphatase 9 in Placenta and Its Relationship with Gestational Diabetes Mellitus

**DOI:** 10.1155/2019/1963178

**Published:** 2019-10-20

**Authors:** Qiang Wei, Xiaomin Pu, Li Zhang, Yi Xu, Meifan Duan, Yuemei Wang

**Affiliations:** Department of Obstetrics and Gynecology, Key Laboratory of Birth Defects and Related Diseases of Women and Children of the Ministry of Education, West China Second Hospital, Sichuan University, Chengdu, China

## Abstract

**Introduction:**

The aim of the present study was to examine placental levels of DUSP9 mRNA and protein and to investigate the potential role of DUSP9 in the development of gestational diabetes mellitus (GDM).

**Methods:**

Placental tissues from pregnant women with GDM (*n* = 17) and normal healthy pregnant women (*n* = 16) were collected at delivery. The expression of DUSP9 mRNA in placental tissue was analyzed by real-time PCR, while the expression of DUPS9 protein was evaluated by immunohistochemistry and western blot. Differences in the expression levels of DUSP9 mRNA and protein between the two groups were assessed, as well as potential correlations between DUSP9 mRNA expression levels and relevant clinical indicators.

**Results:**

Blood glucose levels were significantly higher in the GDM group than in the control group, based on an oral glucose tolerance test. DUSP9 protein was expressed in the placental cytotrophoblasts in both groups, and placental levels of DUSP9 protein and mRNA were significantly higher in women with GDM. Placental DUSP9 mRNA levels in all 33 women correlated moderately with delivery gestational week (*R* = 0.465, *P* = 0.006), fasting plasma glucose (*R* = 0.350, *P* = 0.046), 1-hour postload plasma glucose (*R* = 0.363, *P* = 0.038), and 2-hour postload plasma glucose (*R* = 0.366, *P* = 0.036), but not with maternal age, preconception body mass index, prenatal body mass index, or neonatal birth weight. Multiple linear regression analysis indicated that delivery gestational week was an influence factor of DUSP9 mRNA levels (*β*_1_ = 0.026, *P* < 0.05).

**Conclusions:**

DUSP9 upregulation in the placenta of GDM pregnant women may promote insulin resistance, which may correlate with the occurrence of GDM. But there is still possibility that DUSP9 upregulation was the results of insulin resistance and/or hyperglycemia. Further research is needed to explore the role of DUSP9 in GDM.

## 1. Introduction

Gestational diabetes mellitus (GDM) refers to abnormal glucose metabolism during pregnancy and is associated with adverse maternal and neonatal outcomes [1]. The prevalence of GDM is around 8.9-53.4% of pregnancies and depends on the prevalence of type 2 diabetes mellitus in the general population [2]. Similar to type 2 diabetes mellitus, insulin resistance plays an important role in GDM [3, 4]. Blood glucose metabolism and insulin resistance depend on two main pathways [5, 6]. In the phosphatidylinositol-3-OH kinase (PI-3K) pathway, insulin binds to alpha subunits of the insulin receptor (INSR) on the surface of the cell membrane, leading to activation of tyrosine kinase beta subunits in the cytoplasm, which phosphorylate insulin receptor substrate 1 (IRS-1) tyrosine bound to the beta subunits [5, 6]. Phosphorylated IRS-1 further activates PI-3K and initiates a series of downstream signaling cascades. It stimulates glycogen synthesis by promoting the phosphorylation of glycogen synthase kinase-3 (GSK-3) and serine/threonine protein phosphatase 1 (PP1), and it also promotes the translocation of intracellular glucose transporter 4 (GLUT4) vesicles to the cell membrane, increasing glucose intake and lowering the concentration of blood glucose [5, 6]. The PI-3K pathway mediates the metabolic effects of insulin, including the transcription of insulin-regulated genes. In the second pathway, phosphorylated IRS-1 activates Ras protein, and the downstream Ras direct effector factors Raf and MAPK are activated in cascade. MAPK is further regulated by extracellular signal-regulated kinase (ERK), c-Jun NH2-terminal kinase (JNK), and P38MAPK to promote cell growth and proliferation and regulate blood glucose metabolism [7]. Activated mitogen-activated protein kinase (MAPK) can also exert negative feedback on the PI-3K signaling pathway to regulate blood glucose metabolism [7, 8]. Dual-specificity phosphatase 9 (DUSP9), a tyrosine-specific phosphatase, is inactivated by dephosphorylation of MAPK, and it negatively regulates the MAPK cascade [7]. IRS-1 is a key protein in the insulin signaling pathway involved in the regulation of blood glucose metabolism [5, 6].

DUSP9 regulates insulin resistance by affecting IRS-1-related signaling pathways [5, 9, 10]. Several genome-wide association studies have identified DUSP9 as a susceptibility gene for type 2 diabetes mellitus, in particular the DUSP9 rs5945326 polymorphism [3, 11, 12]. DUSP9 is highly expressed in the placenta, where it is evident in villous trophoblast and its levels decline during development [13]. DUSP9 is important for placental development, and its deletion can cause placental dysfunction and embryonic death [14]. DUSP9/MKP-4, while dispensable for mammalian embryonic development, is essential for placental organogenesis, highlighting the critical role of dual-specificity MAPK phosphatases in the regulation of developmental outcomes in vertebrates [14].

The present study further explored the relationship between DUSP9 and the pathogenesis of GDM by comparing the expression of DUSP9 mRNA and protein in placental tissues of pregnant women with or without GDM and by correlating DUSP9 expression levels with clinical characteristics.

## 2. Material and Methods

### 2.1. Patient Selection

All pregnant women included in the study followed regular prenatal care and had a full-term cesarean delivery at the Department of Obstetrics, West China Second Hospital of Sichuan University, from January to December 2017. All women underwent a 75 g oral glucose tolerance test (OGTT) from 24 to 28 weeks of gestation. A total of 33 patients were included: 17 with GDM, as diagnosed based on the World Health Organization diagnostic criteria from 2013 [5], and 16 without any pregnancy complications as a control group.

Exclusion criteria for both groups were as follows: (i) multiple pregnancies, (ii) preterm delivery, (iii) other complications (e.g., confirmed type 2 or type 1 diabetes mellitus, hypertensive disorder, intrahepatic cholestasis of pregnancy, placenta previa, placental abruption, and pregnancy with heart disease), (iv) having a history of organ transplantation and immunotherapy, or (v) taking drugs that might interfere with glucose and lipid metabolism during pregnancy (such as indomethacin, phentolamine, furosemide, phenytoin sodium, and cortisone).

The study protocol was approved by the ethics committee of West China Second Hospital of Sichuan University, and participants gave written informed consent to be enrolled.

### 2.2. Placental Collection

Immediately after the delivery of the placenta, several samples (each 1 × 1 × 1 cm) of fresh villus tissue were collected in tubes while avoiding the calcification and infarction in the center of the maternal surface. A gauze was used to absorb the blood. Sample specimens were numbered and quickly preserved in liquid nitrogen. At the same time, several villus tissue samples (2 × 1 cm) were collected and repeatedly washed with physiological saline solution to remove blood stains. Excess water was removed with filter paper, and samples were fixed with 10% formaldehyde for the preparation of paraffin blocks.

### 2.3. Immunohistochemistry

Paraffin sections were prepared according to previously published methods [15]. The expression of target proteins in the sections was then measured by immunohistochemistry using the following procedure. Sections were subjected to an antigen retrieval method using citrate buffer at 98°C for 15 min. After the endogenous peroxidase was inactivated by soaking in 3% hydrogen peroxide at 37°C for 15 min, sections were incubated with the primary antibody against DUSP9 (1 : 100 dilution; Proteintech, Chicago, USA) at 37°C for 60 min, followed by the secondary antibody horseradish peroxidase-labeled polymer-conjugated goat anti-rabbit immunoglobulin G (IgG; DakoCytomation, USA) at 37°C for 45 min. Peroxidase activity was detected by incubating sections with DAB substrate, producing a brown stain. Sections were counterstained with hematoxylin. The results were observed under a light microscope (Olympus, Tokyo, Japan). All slides were stained on the same day using the same solutions in order to minimize variations. As negative controls, tissue sections were incubated in parallel with isotype IgG at the same concentration as the primary antibody or with only secondary antibody without primary antibody.

### 2.4. Real-Time PCR

Total RNA of fresh frozen villus tissue was extracted with TRIzol reagent (Invitrogen, Carlsbad, CA) according to the manufacturer's instructions. The extracted RNA was reverse-transcribed into cDNA using the Return Aid™ First Strand cDNA Synthesis Kit (MBI, Lithuania) and then analyzed by real-time PCR. The primers for DUSP9 were ATCCGCTACATCCTCAATGTCA (forward) and GGGACAAGGCCTCATCAATGAA (reverse), and they yielded an amplicon of 157 bp. Primers for ACTB were GAAGATCAAGATCATTGCTCCT (forward) and TACTCCTGCTTGCTGATCCACA (reverse), and they yielded an amplicon of 111 bp. Each reaction (30 *μ*L) was prepared with 5 *μ*L cDNA template, 1 *μ*L of each primer (10 *μ*M), 2 *μ*L of 10 *μ*M probe, 0.25 *μ*L of 25 mM dNTPs, 0.2 *μ*L of 5 U/*μ*L Taq enzyme, 3 *μ*L of 25 mM magnesium chloride, 3 *μ*L of 10x buffer, and 14.55 *μ*L of double-distilled water. The reaction was carried out on a FTC2000 real-time quantitative PCR system (Funglyn Biotech, Toronto, Canada). Amplification conditions were as follows: after the initial denaturation at 94°C for 2 min, samples were subjected to 45 cycles of denaturation at 94°C for 20 s, annealing at 54°C for 20 s, and elongation at 72°C for 30 s. The fluorescence acquisition time of the PCR instrument was set to equal the extension period of the amplification reaction. At the end of the 45-cycle amplification reaction, the system analyzed the fluorescence intensity growth index of each reaction tube in each cycle reaction and plotted the corresponding amplification kinetic curve. The expression levels of mRNA were standardized against those of beta-actin.

### 2.5. Western Blotting

Placental samples (30 mg) were homogenized in ice-cold lysis buffer (7 M urea, 2 M thiourea, 4% CHAPS, 2 mM TBP, 50 mM DTT, 2 mM PMSF, 1 mg/mL DNase I, 0.2 mg/mL RNase A) and centrifuged at 12,000 *g* for 10 min at 4°C, and then, the supernatants were collected and assayed for total protein concentration using the BCA kit (Beyotime Biotech, Shanghai, China). Equal amounts of protein were separated by sodium dodecyl sulfate polyacrylamide gel electrophoresis (SDS-PAGE) and transferred onto polyvinylidene difluoride membranes at 60 V for 60 min. Membranes were blocked for 60 min at room temperature in Tris-buffered saline. The blots were incubated overnight at 4°C with primary antibodies against DUSP9 (1 : 300 dilution; Proteintech) and beta-actin (mouse anti-human, monoclonal; Bioss Biotech, Beijing, China). Membranes were washed three times with washing buffer and incubated with horseradish peroxidase-labeled goat anti-rabbit IgG (1 : 20000; Zhongshan Jinqiao Biotech, Beijing, China) at 37°C for 1 h. Protein band intensities were quantified using Quantity One 1-D Analysis Software (Bio-Rad Laboratories, USA).

### 2.6. Statistical Analysis

Data were analyzed using SPSS 22.0 (IBM, Armonk, NY, USA). Intergroup differences were assessed for significance using the *t*-test when the data were normally distributed and showed homogeneity of variance; otherwise, differences were assessed using the Mann–Whitney *U* test. Pearson's correlation was used to analyze the correlation between mRNA expression and clinical data. Graphs were designed using GraphPad Prism software 5 (GraphPad Software, La Jolla, CA, USA). Differences were considered statistically significant if the two-tailed *P* < 0.05.

## 3. Results

### 3.1. Patient Characteristics

All the included pregnant women followed regular prenatal care in our hospital and were given medical nutrition guidance and weight management during pregnancy. All pregnant women had normal weight gain during pregnancy. In addition, pregnant women with GDM were given further diet, regular exercise, and blood glucose monitoring. Among them, nine patients have received GDM therapy (diet and regular exercise) and eight patients have received GDM therapy (diet, regular exercise, and insulin) until the delivery and their blood glucose levels have been well-controlled. There were no significant differences in maternal age, preconception body mass index (BMI), prenatal BMI, delivery gestational age, or neonatal birth weight between GDM mothers and the control group (Table 1). The 75 g OGTT three-point blood glucose levels (fasting and 1-hour and 2-hour postload glucose) were significantly higher in the GDM group than in the control group (all *P* < 0.001; Table 1).

### 3.2. Expression of DUSP9 Protein in Placental Tissue

**Figure 1:**
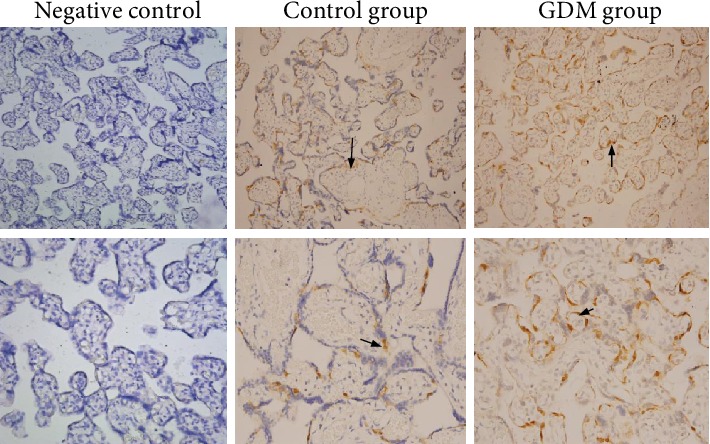
Immunohistochemistry showing the localization of DUSP9 protein in placental tissues from mothers with gestational diabetes mellitus (GDM) or with normal pregnancies. As negative controls, tissue sections were incubated in parallel with isotype IgG at the same concentration as the primary antibody or with only secondary antibody without primary antibody. DUSP9 was observed in the cytotrophoblasts of both groups (arrows). Magnification: 200x (upper row) or 400x (lower row).

DUSP9 protein in placental tissues was detected by immunohistochemistry in a subset of five GDM and four control women. In both groups, the protein was observed in the cytoplasm of cytotrophoblasts (Figure 1).

**Figure 2:**
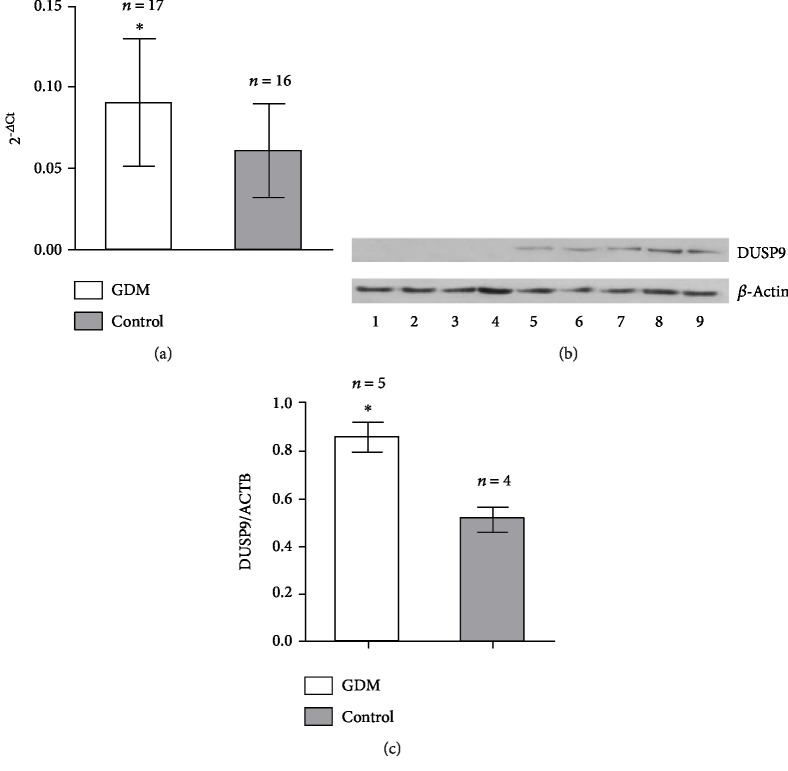
Levels of DUSP9 protein and mRNA in placentas from women with gestational diabetes mellitus (GDM) or normal pregnancies. (a) Real-time RT-PCR showed the expression of DUSP9 mRNA in placental tissues of GDM group was significantly higher than that of the control group (*P* < 0.05). (b) DUSP9 protein levels in placenta tissue based on western blot. Lanes 1-4, four control women; lanes 5-9, five GDM women. Quantitation of the blot results is shown. (c) The expression of DUSP9 protein in placental tissues of the GDM group was significantly higher than that of the control group (*P* < 0.05).

Western blotting was used to compare placental levels of DUSP9 protein in the same subset of five GDM and four control women as examined by immunohistochemistry. DUSP9 levels were significantly higher in the GDM group (mean gray value, 0.85 ± 0.06 vs 0.51 ± 0.05, *P* < 0.001; Figures 2(b) and 2(c)).

### 3.3. Levels of DUSP9 mRNA in Placental Tissue

Real-time reverse transcription PCR was used to compare levels of DUSP9 mRNA in placental tissues from the entire GDM group (*n* = 17) and control group (*n* = 16). Levels of mRNA were significantly higher in the GDM group (0.09 ± 0.04 vs. 0.06 ± 0.03; *t* = 2.321, *P* = 0.027; Figure 2(a)).

### 3.4. Correlation of DUSP9 mRNA Levels in Placenta and Clinical Indicators

**Figure 3:**
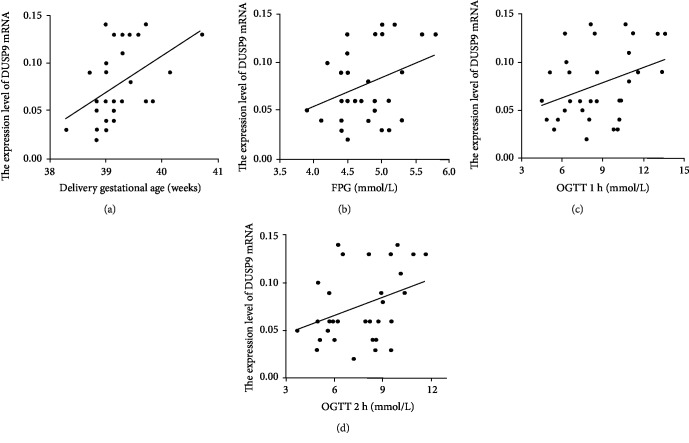
Potential correlation of DUSP9 mRNA levels in the placenta with (a) gestational week of delivery, (b) fasting plasma glucose (FPG), (c) 1-hour postload plasma glucose in an oral glucose tolerance test (OGTT), or (d) 2-hour postload plasma glucose.

Across all 33 women in the study, DUSP9 mRNA levels in the placenta showed a moderate, positive correlation with gestational week of delivery and 75 g OGTT three-point blood glucose levels (Table 2, Figure 3). There was no correlation with maternal age, preconception BMI, prenatal BMI, or neonatal birth weight. Multiple linear regression analysis using delivery gestational week and 75 g OGTT three-point blood glucose level as independent variable and DUSP9 mRNA levels as the dependent variable indicated that delivery gestational week was independently an influence factor of DUSP9 mRNA levels (*β*_1_ = 0.026, *P* < 0.05) (Table 3).

## 4. Discussion

In this study, we examined placental expression of DUSP9 mRNA and protein and its potential role in the development of GDM. We found that DUSP9 protein was expressed in the placental cytotrophoblasts in both groups and placental levels of DUSP9 protein and mRNA were significantly higher in women with GDM. Placental DUSP9 mRNA levels in all 33 women correlated moderately with fasting plasma glucose (*R* = 0.350, *P* = 0.046), 1-hour postload plasma glucose (*R* = 0.363, *P* = 0.038), and 2-hour postload plasma glucose (*R* = 0.366, *P* = 0.036), but not with maternal age, preconception body mass index, prenatal body mass index, or neonatal birth weight. These results suggest that DUSP9 upregulation in the placenta of GDM pregnant women may promote insulin resistance, which may correlate with the occurrence of GDM. But there is still possibility that DUSP9 upregulation was the results of insulin resistance and/or hyperglycemia. Our results with GDM women may provide valuable insights into understanding diabetes in light of the contribution of insulin resistance to both GDM and type 2 diabetes mellitus and in light of results suggesting that increased maternal insulin resistance promotes placental growth and inhibits placental efficiency [4]. Indeed, maternal insulin resistance may contribute to altered placental size and function in pregnancies with obesity and GDM [4].

There are conflicting opinions on whether DUSP9 promotes or inhibits insulin resistance. Studies have suggested that DUSP9 promotes obesity-related insulin resistance [9]; in adipocytes, DUSP9 may promote insulin resistance by inhibiting insulin-stimulated differentiation and glucose uptake [9]. On the other hand, studies have suggested that DUSP9 can inhibit insulin resistance by acting as a MAPK phosphatase and influencing downstream signaling factors of the MAPK pathway, such as ERK, JNK, and p38 [10]. DUSP9 may inhibit insulin resistance by antagonizing the effects of tumor necrosis factor-*α* [10]. Our results suggest that DUSP9 upregulation in the placenta of GDM pregnant women may promote insulin resistance by acting as a MAPK phosphatase and influencing downstream signaling factors of the MAPK pathway, which may correlate with the occurrence of GDM.

Our results linking DUSP9 with GDM are consistent with previous work in which we evaluated the DUSP9 polymorphism at locus rs5945326 in 206 GDM pregnant women and 189 normal pregnant women [16]. Although genotype and allele frequencies were similar between GDM and control women, high-density lipoprotein levels were significantly higher in women with the GG genotype than in those with AG or AA genotypes. This suggests that the G allele may protect against glucolipid metabolism, in agreement with a genome-wide association study (GWAS) [16]. More broadly, many GWAS in different ethnic groups have identified DUSP9 as a susceptibility gene for type 2 diabetes mellitus [3, 11, 12]. In particular, the A allele at the locus rs5945326 may increase risk of the condition, while the G allele may protect against it [3, 11, 12].

Studies have shown that DUSP9 is abundantly expressed in placental tissues and in adult kidney, testis, and adipose tissue, as well as in embryonic liver [14, 17]. However, we are unaware of studies on the expression of DUSP9 gene in placenta and its correlation with GDM. The present results show that DUSP9 is expressed in placental trophoblast cells and that levels of DUSP9 mRNA and protein in placental tissues are significantly higher in pregnant women with GDM than in normal pregnant women. In addition, our results showed that the expression level of DUSP9 mRNA moderately correlated with the gestational week of delivery and the 75 g OGTT three-point blood glucose levels. Furthermore, when using a multiple linear regression, we found that delivery gestational week was an influence factor of DUSP9 mRNA levels.

In summary, this study showed that levels of DUSP9 mRNA and protein were significantly higher in the placenta of GDM pregnant women than in normal pregnant women. Levels of DUSP9 mRNA moderately correlated with blood glucose levels. We hypothesize that DUSP9 upregulation in trophoblast cells promotes insulin resistance in the pathogenesis of GDM. But there is still possibility that DUSP9 upregulation was the results of insulin resistance and/or hyperglycemia. Further research is needed to explore the role of DUSP9 in GDM.

## Figures and Tables

**Table 1:** Characteristics and clinical indicators of mothers with gestational diabetes mellitus (GDM) or healthy pregnancies.

Characteristic	GDM group (*n* = 17)	Control group (*n* = 16)	*P*
Maternal age (years)	33.24 ± 3.82	31.06 ± 2.59	0.067
Preconception BMI (kg/m^2^)	21.86 ± 2.39	20.69 ± 2.32	0.166
Prenatal BMI (kg/m^2^)	26.54 ± 3.21	25.81 ± 2.74	0.491
Gestational age at delivery (weeks)	39.30 ± 0.54	39.08 ± 0.29	0.167
Neonatal birth weight (g)	3259 ± 438	3299 ± 349	0.777
FPG (mmol/L)	4.98 ± 0.40	4.46 ± 0.27	*<0.001*
OGTT 1 h (mmol/L)	10.66 ± 1.58	6.54 ± 1.23	*<0.001*
OGTT 2 h (mmol/L)	9.28 ± 1.24	5.91 ± 1.18	*<0.001*

Values are expressed as *mean* ± *SD*. Abbreviations: BMI: body mass index; FPG: fasting plasma glucose; GDM: gestational diabetes mellitus; OGTT: oral glucose tolerance test.

**Table 2:** Correlation of DUSP9 mRNA levels in the placenta with clinical indicators relevant to gestational diabetes mellitus.

Indicator	Pearson's correlation	
	*R*	*P*
Maternal age (year)	0.028	0.878
Preconception BMI	0.106	0.557
Prenatal BMI	0.077	0.671
Delivery gestational week	*0.465*	*0.006*
Neonatal birth weight	0.124	0.491
FPG	*0.350*	*0.046*
OGTT 1 h	*0.363*	*0.038*
OGTT 2 h	*0.366*	*0.036*

Abbreviations: BMI: body mass index; FPG: fasting plasma glucose; OGTT: oral glucose tolerance test.

**Table 3:** Multiple linear regression analysis of factors associated with DUSP9 mRNA levels.

	*β*	*P*
Constant	-1.267	0.023
Delivery gestational week	0.032	*0.026*
FPG	0.009	0.591
OGTT 1 h	-0.000087	0.986
OGTT 2 h	0.004	0.476

Abbreviations: FPG: fasting plasma glucose; OGTT: oral glucose tolerance test.

## Data Availability

The data used to support the findings of this study are included within the article.
